# Critical Current Simulation and Measurement of Second Generation, High-Temperature Superconducting Coil under External Magnetic Field

**DOI:** 10.3390/ma11030339

**Published:** 2018-02-26

**Authors:** Dongmin Yu, Huanan Liu, Xinhe Zhang, Taorong Gong

**Affiliations:** 1 Department of Electrical Engineering, Northeast Electric Power University, Jilin 132012, China; d.yu@neepu.edu.cn; 2 China Electric Power Research Institute, Beijing 100192, China; zhangxinhe10@epri.sgcc.com.cn (X.Z.); gongtaorong@epri.sgcc.com.cn (T.G.)

**Keywords:** superconducting coil, critical current, simulation, external magnetic field

## Abstract

This paper studies the critical current of second generation, high temperature superconducting coils under an external magnetic field experimentally and numerically. Two identical coils with different coated conductors are fabricated and tested under a direct current (DC) magnetic field along the axis of the coil. Then, a numerical model in cylindrical coordinates based on a sheet current model is built by taking the measured magnetic field dependency to analyze the current distribution and magnetic field distribution. The simulated critical currents of the coils under the DC magnetic field have good agreement with the measured results. We find that under the in-phase field, the critical current decreases as the magnetic field in the innermost turn is enhanced by the external field. Meanwhile, the anti-phase external field increases the critical current a bit at first, then decreases the critical current. We further discuss the critical current criteria of the coils, showing that the parallel field plays a more important role in critical current determination.

## 1. Introduction

Second generation (2G), high-temperature superconductors (HTS), also known as Rare Earth Barium Copper Oxide (REBCO) tapes, have high critical currents under external magnetic fields; thus, pancake coils wound with REBCO are widely used as insert magnets for high magnetic field devices [1,2,3,4,5]. As such, attentions have been devoted to investigating the properties of these coils [6,7,8]. For high-field applications, the critical current of REBCO coils is one of the key issues. From an engineering point of view, the critical current of the coils is defined as the maximum current that can safely flow in the superconductors [9]. The prediction of critical currents is especially difficult while taking into account the field dependency of the critical current density in the presence of external magnetic fields.

Research on superconducting coils with coated conductors started as early as 2006, when M. Polak et al. measured the magnetic field and alternating current (AC) loss of the REBCO coil [10]. In the following year, H. Fukushima et al. fabricated four Gadolinium Barium Copper Oxide (GdBCO) coils and measured the critical current in the self-field [11]. Their experiments showed that the critical current was determined by the magnetic field with 45° to the *c*-axis of the GdBCO layer in the middle of the coil edge. In 2009, J. Souc et al. measured the critical current of Yttrium Barium Copper Oxide (YBCO) coil under a self-field with different turns, and these measurements had good agreement with the numerical results [12]. M. Chudy rotated the racetrack coil under a DC field and analyzed the critical current with different angles [13]. Recently, D. Hu et al. characterized an epoxy-impregnated, triangle-shape superconducting coil [14]. Although intensive research has been conducted on the critical current of 2G HTS coils, little attention has been paid to the possible influence of an external DC field.

Another way to investigate the coil is by employing analytical and numerical methods. Numerous studies have been performed [7,15,16,17,18,19,20] and some powerful tools are available. However, almost all these models are used to simulate the superconducting coil in the self-field. Reference [21] proposed a theoretical model to calculate the critical current under an external parallel field, assuming uniform current distribution across the superconducting region. However, only M. Zhang et al. simulated the current distribution under a DC field to predict the critical current of the coil using the finite element method with the H formula [9].

Up to now, the critical current characterization of superconducting coil with a coated conductor under a DC magnetic field has rarely been investigated. Further understanding of the critical currents of coils is crucial for future engineering applications of superconducting coils. This paper aims to study the critical current of 2G HTS coils under a DC magnetic field parallel to the axis of the coil.

The paper is organized as follows: Two superconducting coils with coated conductors from different suppliers were wound and tested to characterize the critical current under different external fields in Section 2. A numerical model was established using sheet current line models, incorporating the measured magnetic field dependency of the critical current in Section 3. In Section 4, the measured and simulated results were compared to validate the model. We further compared the critical currents of coils with different field dependencies. The main contributions of this paper can be summarized as follows: (1) A new numerical model based on sheet current approximation in cylindrical coordinates is proposed to simulate the electromagnetic field of YBCO coils. This model speeds up the simulation by ignoring the micrometer-level meshes; (2) This paper, for the first time, experimentally investigates YBCO tapes with a special field dependency that critical current degradation dominates by a parallel magnetic field.; (3) We further compare the tapes with different field dependencies of the critical current for coil application, and the results show that the parallel field plays a more important role in critical current determination.

## 2. Experimental Setup

**Figure 1:**
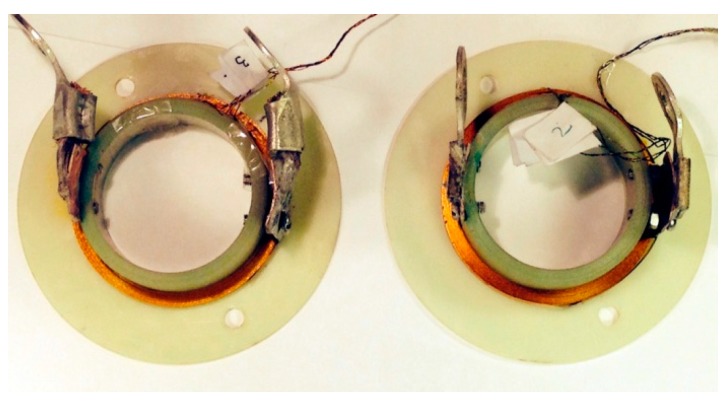
The 20-turn coils: (**left**) SuNAM coil; (**right**) SuperPower coil.

Two pancake coils with 20 turns were prepared, as shown in Figure 1. One coil was made from HCN4045-coated conductor from SuNAM. The other coil was wound with SCS4050 tape from SuperPower. Both coils had the same geometry and winding method. The coils were wound on a G10 tube with a diameter of 56 mm using a winding machine under constant tension. Two small voltage taps were soldered on both ends of the coil to measure the voltage drop along the whole coil, which is denoted as E-I curve. This E-I curve is used to determine the critical current of the coil. Another two voltage taps were soldered on the fifth turn and 15th turn, respectively, to measure the voltage drop of the inner five turns and the outer five turns. The labels in Figure 1 are used to identify the voltage taps. A detailed list of parameters for the coils is shown in Table 1.

**Figure 2:**
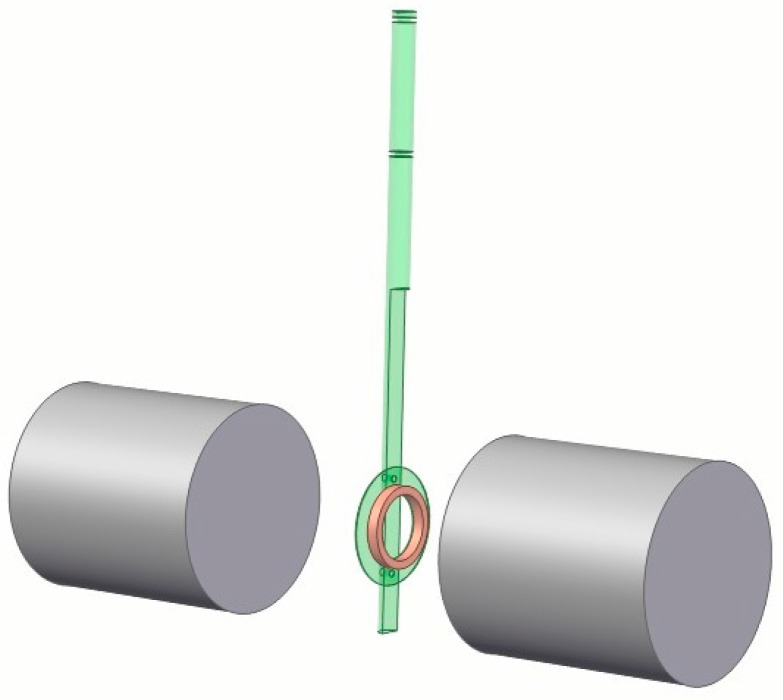
Schematic view of the experimental setup; the cylinders represent the magnet poles. The coil is fixed by a non-magnetic nut in the center of the two magnet poles.

The magnetic field experimental setup is presented in Figure 2 schematically. The pancake coil was fixed in the gap between two poles of a magnet. The coil and the rod were connected by non-magnetic nuts. The axis of the coil was along the direction of the magnetic field; thus, the coil was parallel to the external parallel field. It was challenging to ensure a homogeneous field in the rather large gap between the poles of the magnet. For this purpose, the position of the coil was carefully aligned to the axis of the cylindrical poles. Furthermore, the circumferential fields along the coil were calibrated before the experiments and the field difference was within 5%. This result was sufficiently uniform for our experiments. The maximum permissible current of the electromagnet was 28 A when the field was 0.54 T, with a gap between the poles of 18 cm. The diameter of the magnetic poles was 15 cm.

**Figure 3:**
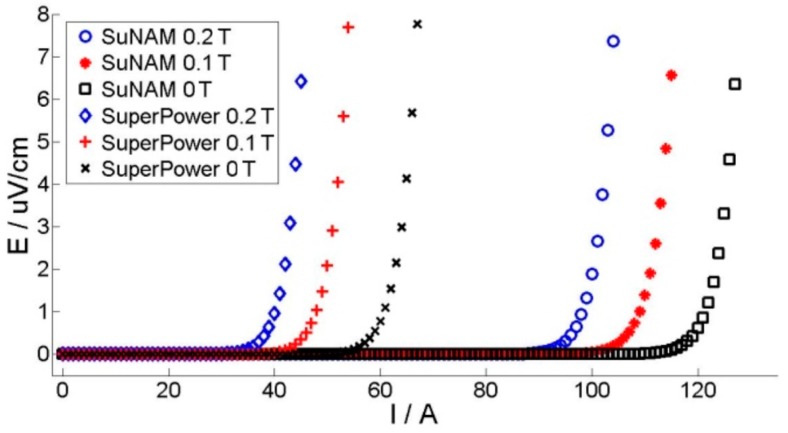
E-I curves under a DC field of 0.2 T, 0.1 T, or 0 T for SuNAM and SuperPower coils.

The liquid nitrogen bath, in which the coils were immersed, was placed between the magnet poles. The simple four-point method was used to measure the critical current of the coil with two voltage taps on both ends. A TDK-Lambda DC power supplier (Devon, UK) was used as a current source and the voltages were acquired by an NI SCXI 1328 card (Buckinghamshire, UK). The measurement setup was controlled by the LabVIEW program (Version 7.0, National Instrument, Austin, TX, USA) [22]. Figure 3 shows the E-I curves of both coils under different fields.

**Figure 4:**
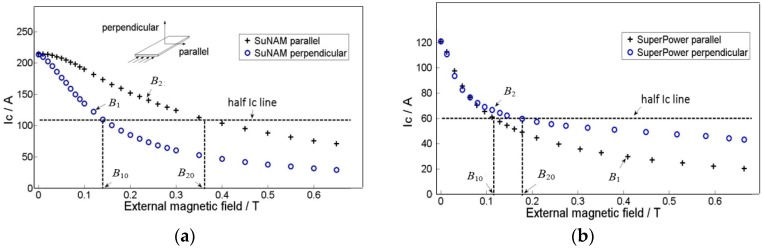
Measured field dependency for SuNAM tape and SuperPower tape. (**a**) *B*_1_ represents the lower curve and *B*_10_ is the field when the current is half of the critical current on the *B*_1_ curve; (**b**) *B*_2_ represents the upper curve and *B*_20_ is the field when the current is half of the critical current on the *B*_2_ curve.

The field dependencies of critical currents for the tapes at 77 K were also measured, as shown in Figure 4. The critical currents of the tapes vary with both the magnitude of the magnetic field and the direction of the magnetic field. In the rest of this paper, the perpendicular field is noted as *B*_r_, which is perpendicular to the surface of the conductor. Similarly, the parallel field is denoted as *B*_z_. These two graphs reveal that the SuperPower is quite different from the SuNAM tape in the magnetic field; that is, critical current of SuperPower tape in the parallel field is smaller than that under the perpendicular field, while normal tapes have a smaller critical current under the perpendicular field, such as the SuNAM tape. According to the 1 μV/cm criterion, the SuNAM tape has a critical current of 217 A in the self-field, while the SuperPower tape has a critical current of 120 A.

Anisotropy of the YBCO tape under an external magnetic field would greatly influence the current and magnetic field distribution inside the coil. Figure 4 presents the measured critical current in perpendicular and parallel directions under different external fields. The perpendicular field has a direction parallel to the *c*-axis of the superconductor plane. In this paper, we use a modified Kim model to consider the anisotropy of YBCO tapes in the simulation. The method avoids the complicated optimization process of variables determination. The modified Kim model is expressed by the following formula [17]:

(1) $$J_{c}\left(B_{1},B_{2}\right)=\frac{J_{c0}B_{10}}{B_{10}+\sqrt{{\left(B_{1}\right)}^{2}+{\left(kB_{2}\right)}^{2}}}$$

Here, *B*_1_ is the lower curve of each tape in Figure 4 and *B*_2_ is the higher curve. *B*_10_ is the field when the current is half of the critical current on the *B*_1_ curve; similarly, *B*_20_ is the corresponding field on the *B*_2_ curve. *k* is the ratio between *B*_10_ and *B*_20_, which describes the anisotropy of *B*_1_ and *B*_2_. All the data we use are based on the measurement of SuNAM tape and SuperPower tape at 77 K. Table 2 summarizes the derived values from the measured data.

## 3. Simulation Model

**Figure 5:**
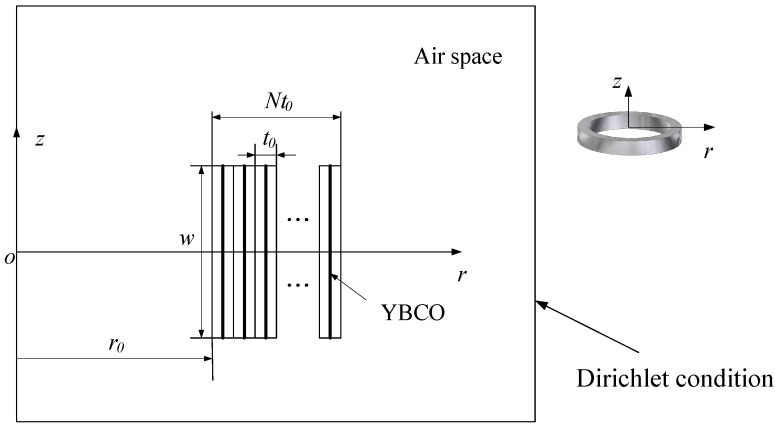
A coil with N tapes: the width is *w* and the thickness is *t*_0_. The radius of the coil is *r*_0_. YBCO superconductor layers are presented by thicker lines.

The YBCO pancake coil model based on the sheet current model is implemented in the software MATLAB (2015a, MATLAB, Natick, MA, USA). This method has the advantage of good efficiency, because only the superconducting tapes are meshed when solving the highly non-linear current distribution. Figure 5 shows the cross-section view of a superconducting coil with YBCO coated conductors. The thin YBCO layers are depicted as black lines in the center. The YBCO tape has several layers, including substrate, stabilizer, and buffer layers. We only model the superconducting layer, assuming that all other space is occupied by air. The gap between the tapes, denoted as *t*_0_, is determined using the tape thickness, and presented in Table 1. Each YBCO layer represents one turn of the pancake coil; the innermost turn is Turn 1, while the outmost turn is Turn 20. Applied current *I*_app_ is assigned to each YBCO layer. The superconducting lines are evenly meshed with 50 line elements, while the air region is meshed by triangle elements.

**Figure 6:**
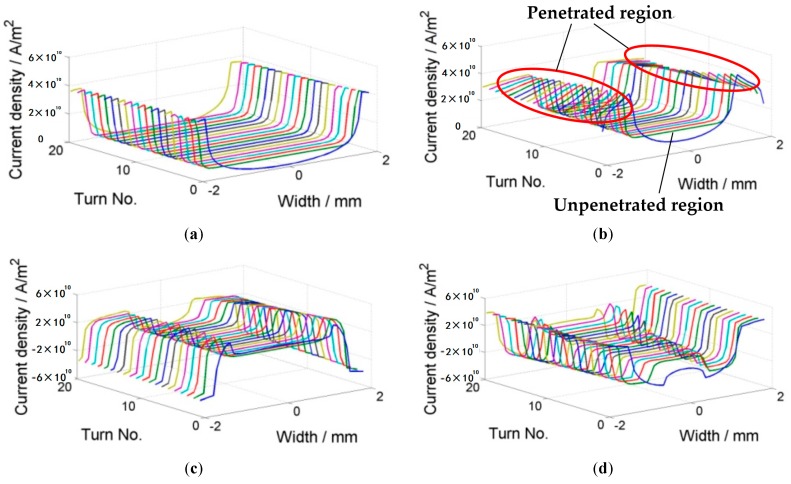
Current distribution for 20 turns with an external field of −0.2 T: (**a**) ramping up to 30 A; (**b**) ramping up to 80 A; (**c**) ramping down to −30 A; (**d**) ramping up to 0 A. Tape No. 1 is in the innermost turn.

The problem of superconducting simulation is to find the current distribution that is equivalent to implicit optimization problem through variational theory [18]; in other words, to find *J*_i_:

(2) $$\underset{i\in \Omega }{\sum }\left(\frac{1}{2}A_{i}^{m}+f_{i}^{m}+\tau C_{j}\right)J_{i}S$$

for each tape:

(3) $$\int_{-w/2}^{w/2}J_{i}dz=I_{\mathrm{app}}$$

for all the elements:

(4) $$J_{i}\leq J_{c}\left(B_{r},B_{z}\right)$$

where *Ω* is the set of all elements, *A_i_* and *J_i_* are the vector potential and current in the circumferential direction in the *i*th element, $f_{i}^{m}=-A_{i}^{m-1}$, *C_j_* is the voltage due to the source in the *j*th tape, and $J_{c}\left(B_{r},B_{z}\right)$ is the critical current density with field dependencies. Thus, Equation (3) means that each tape transports the current of *I*_app_ and Equation (4) represents the Bean model [18]. This equation can be optimized by iterations, and it is implemented by using the constrained quadratic minimization procedure in MATLAB. The calculation above only gives the current distribution in the tapes that is assigned to each YBCO layer. The field within and around the coil is solved using the two-dimensional (2D) finite element method (FEM) in cylindrical coordinates. The coil geometry is shown in Figure 5, assuming that the air domain is so large that the magnetic field produced by the applied current decays to zero on the boundary for FEM. A varying current with a sine shape is applied to the coil and it peaks at 80 A. The frequency of the applied current is 1 Hz. Figure 6 shows the current distribution within the coil for four steps: 30 A, peaking current 80 A, −30 A, and 0 A. Each line represents one tape and the *z*-axis is the magnitude of the current density. From Figure 6b, we can clearly see two regions in the superconducting region: the penetrated region and the unpenetrated region. They are also called the critical region and the subcritical region, consistent with the assumption of Clem [16]. The penetrated region expands from both ends into the central region. In the penetrated region, the current density is bounded by the critical current density. As it was previously pointed out that the critical current decreases in the external field, we can see a clear slope of the current density in the penetrated region. In Figure 6d, the total applied current in each tape is zero; however, we can see that the current density is not homogenously zero. On the contrary, there are both positive and negative currents within the region. This shows the hysteresis nature of the superconducting coil after magnetization.

**Figure 7:**
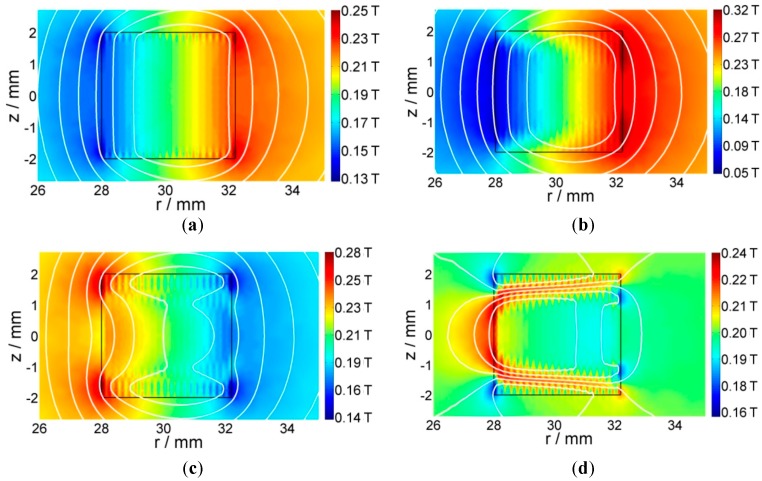
Magnetic field distribution and flux line plot of a 20-turn coil with an external field of −0.2 T. The white contour lines represent the directions. (**a**) Ramping up to 30 A; (**b**) ramping up to 110 A; (**c**) ramping down to −30 A; (**d**) ramping up to 0. The rectangle outlined in black represents the region occupied by superconductors.

The magnetic field and flux line of the same time step are presented in Figure 7. The color represents the magnitude of the magnetic flux density, while the contours show the isoline of the vector potential. According to the magnetic field contours and directions, we can see that the strong magnetic field locates in the outermost turn, which has a smaller critical current density, as shown in Figure 6. We can also see that *B*_z_ exists only in the subcritical region (magnetic fields are parallel to the tape width direction). This is due to the intrinsic property of the superconductor, which expels the magnetic field. This is consistent with W. Yuan’s papers [17,20], which solve the electromagnetic field through the minimization of the (*B*_r_)^2^ within the subcritical region.

## 4. Discussion

### 4.1. Model Validation

**Figure 8:**
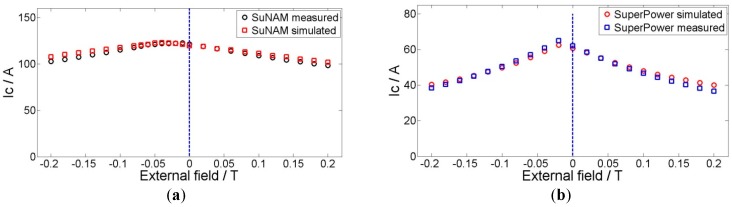
Comparison between measured and simulated critical current for SuNAM (**a**) and SuperPower (**b**) coil with 20 turns in different external fields. The measured critical currents are defined by 0.5 μV/cm in E-I curves.

**Figure 9:**
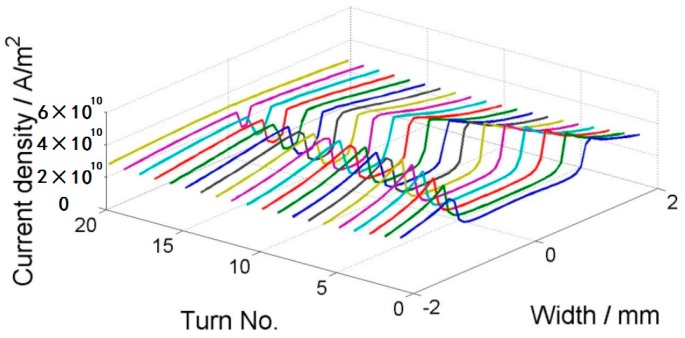
The current distribution when the applied current is 107.5 A and the external field is anti-phase 0.2 T.

For the experimental results, the critical current is usually defined before the voltage reaches certain values, such as 1 μV/cm, 0.5 μV/cm, and 0.1 μV/cm in the measured E-I curve [23]. Figure 3 presents the typical E-I curves, which can be drawn when ramping up the current within the superconducting coils continuously. In this paper, we select the criterion of 0.5 μV/cm to define the coils’ critical currents, which are shown in Figure 8. For the simulation, the determination process is as follows: a linearly increasing current is applied in each tape, and current distribution is checked in each turn step by step. When any single tape is fully penetrated with a certain applied current, we stop the calculation. This applied current is considered as the critical current of the coil, as shown in Figure 9.

We only study the influence of parallel field Hz on the coil critical current. The in-phase field is defined, while the background field is in the same direction as the self-field of the HTS pancakes and the anti-phase field Hz has the opposite direction to the self-field inside the pancakes. Results are illustrated in Figure 8, wherein we can see that the simulations provide a good estimate of the critical current of the superconducting coils.

Both the experiment and simulation show that the coil critical current decreases linearly when the in-phase external field increases. This is because the background field combined with the self-field reduces its critical current of the innermost turn. However, the situation is quite different for the anti-phase external field. While the magnitude of the anti-phase external field increases, the critical current of the coil increases initially and then decreases. This is because the self-field of the weak turn is cancelled by the external field, thus the total field becomes lower compared to that of the in-phase and self-field conditions. If the magnitude of the anti-phase external field increases further, the critical current begins to decrease, as the overall magnetic field decreases the critical current eventually.

**Figure 10:**
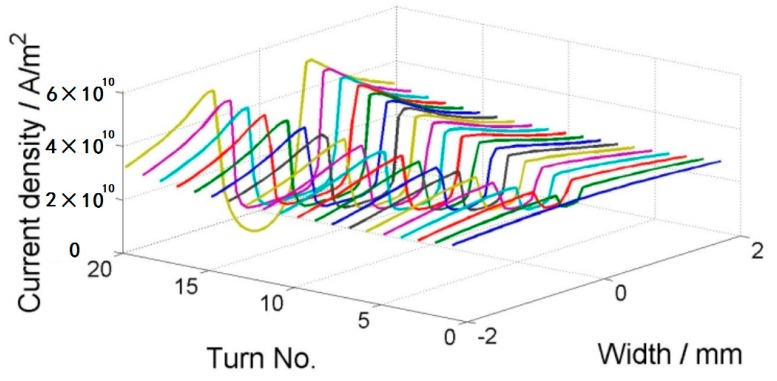
The current distribution when the applied current is 101 A and the external field is in-phase 0.2 T; the inner turn of the SuNAM 20-turn coil is fully penetrated.

**Figure 11:**
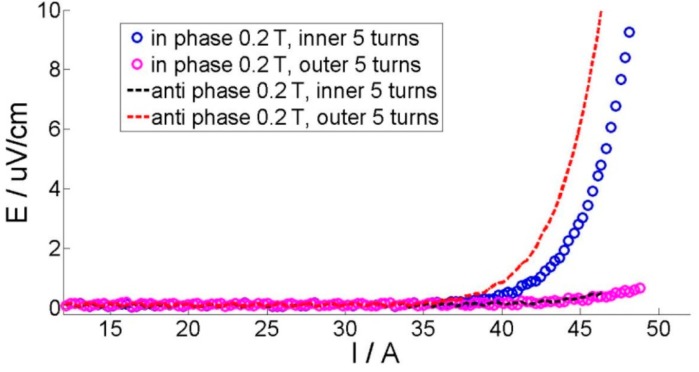
E-I curves of different sections with the SuNAM coil with in-phase and anti-phase 0.2 T magnetic fields.

The existence of the external field also changes the current penetration pattern. Based on the results from the simulation, it is found that under the in-phase field case and the self-field case, the critical current is determined by the innermost turn, which is the first to be fully penetrated, as shown in Figure 10. On the other side, the anti-phase external field shifts the weak turn position from the innermost to the outmost turn when the magnitude of the external field exceeds 0.04 T. This shift can also be verified by measuring the E-I curve. Figure 11 presents the measured voltages on different sections under an external field of −0.2 T. We can see that the voltage within the inner five turns rises earlier than section of the outer five turns under the in-phase field, while the outer five turns section rises earlier under the anti-phase field. This coincides with the simulation results.

### 4.2. Comparison of Two Different Tapes

Recently, some new progress has been made in YBCO tape fabrication by SuperPower, which shows different magnetic field dependencies. The usual tape has a smaller critical current when the external field is perpendicular compared to that which is parallel to the tape, provided that the magnitude is the same. However, this new tape has an opposite dependency, which is that the perpendicular field has larger critical current, as shown in Figure 4. This wire is an AP (Advanced Pinning) type which has zirconium doping that forms BaZrO_3_ nanocolumnar particles in the film. These BaZrO_3_ nanocolumns are aligned in parallel to the *c*-axis of REBCO (Rare Earth Barium Copper Oxide). The result is that the flux pinning for H//c is greatly enhanced. When the pinning from the nanocolumns is strong enough, the *I*_c_ (H//c) becomes higher than the *I*_c_ (H//ab). So, it is of practical interest to analyze the difference when this new SuperPower tape is applied in pancake application under a DC magnetic field compared with a normal magnetic field dependency tape, such as the SuNAM tape. This field dependency is approximated by a modified Kim model, which is shown in Figure 4 and Table 2.

**Figure 12:**
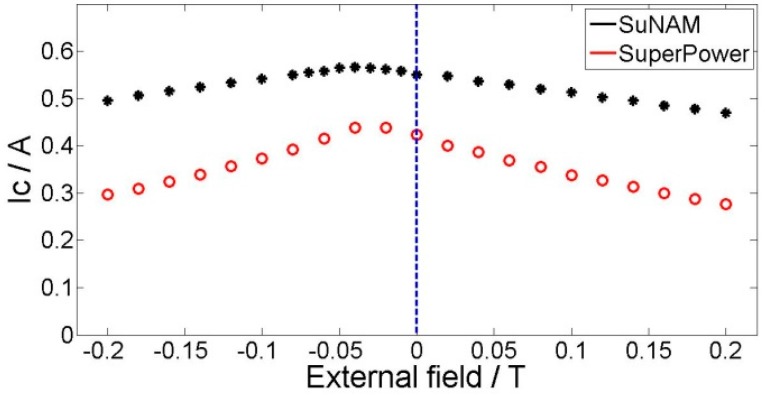
Simulated critical currents of coils after normalization with self-field tape *I_c_*.

We incorporate this new tape into the numerical model and compare the critical current under a DC field with the SuNAM coil presented in Figure 8a. In order to compare the performance of coils consisting of different tapes, both models have the same geometry and the critical current of the tapes are normalized by the critical current under the self-field. Figure 12 shows the simulated critical current under a DC field. Similar trends for the critical current with an external field are found. Specifically, the anti-phase field increases the critical currents first and then decreases it later.

**Figure 13:**
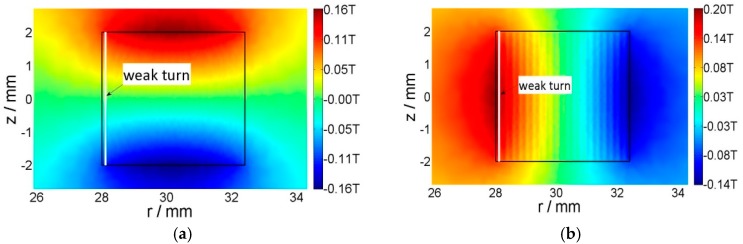
Perpendicular field (**a**) and parallel field (**b**) distribution for 20-turn coil. This coil has a uniform current distribution and each tape transports a current of 100 A.

However, a clear difference is that the SuperPower coil has a lower critical current. Under the self-field, the critical current difference for these two coils with different tapes is 14%, while this difference increases to 18% when the external field is 0.2 T. The reason for this is that the weak turns for these two coils are either the innermost turn, which have large parallel fields. As the SuperPower coil is more sensitive to the parallel field, the critical current is smaller than that of tapes that are less influenced by the parallel field, such as the SuNAM tape. Figure 13 presents the perpendicular field and parallel field of the coil with an applied current of 100 A in the self-field. This graph verifies that the average parallel field is larger than the perpendicular field where the weak turn appears. The existence of a parallel external field decreases the critical current further for the SuperPower coil, which is disadvantageous for its application as an insert coil in high magnetic field devices. The coils are wound with SuNAM and SuperPower tapes, which have different field dependencies. The SuNAM field dependency is presented in Figure 4a and that of SuperPower is shown in Figure 4b.

## 5. Conclusions

Superconducting coils with 2G HTS are widely used as insert magnets for high magnetic field devices, and the prediction of critical currents is especially difficult when taking into account the field dependency of the critical current density under external magnetic fields. This paper studies the critical current of 2G HTS tapes and coils under a parallel DC magnetic field experimentally and numerically. Two types of commercial superconductors with different magnetic field dependencies on the critical current are used to wind the coils, and their critical currents are measured under in-phase and anti-phase external magnetic fields. The numerical model utilizes the minimization energy approach combined with the sheet current model in cylindrical coordinates, in addition to the finite element model to solve the magnetic field. It is found that, contrary to the normal criterion of 1 μV/cm, 0.5 μV/cm is more reasonable to determine the critical currents of the coils. Our results also show that the parallel field plays a more important role in critical current determination.

## Figures and Tables

**Table 1:** Specifications of the coils.

Tape Yype	HCN4045	SCS4050
Manufacturer	SuNAM	SuperPower
Tape *I*_c_	217 A	120 A
Tape thickness	0.20 mm	0.20 mm
Inner diameter	56 mm	
Total turns	20	
Tape width	4 mm	
Insulation	Kapton tape	

**Table 2:** Field dependency parameter for SuNAM and SuperPower tapes.

Type of Tapes	SuNAM	SuperPower
*B*_1_	Perpendicular	Parallel
*B*_10_ (T)	0.140	0.115
*B*_2_	Parallel	Perpendicular
*B*_20_ (mT)	0.360	0.180
*k*	0.140/0.360	0.115/0.180
